# Percutaneous Management of Iatrogenic Aortocoronary Dissection Complicating Diagnostic Angiography or Percutaneous Coronary Intervention

**DOI:** 10.5935/abc.20170105

**Published:** 2017-09

**Authors:** Liang Tang, Xin-qun Hu, Jian-jun Tang, Sheng-hua Zhou, Zhen-fei Fang

**Affiliations:** Department of Cardiology - The Second Xiangya Hospital of Central South University, Changsha, China

**Keywords:** Percutaneous Coronary Intervention, Aortocoronary Dissection, Coronary Artery Bypass, Complications

## Introduction

Aortocoronary dissection is an infrequent, yet potentially catastrophic complication of coronary angiography (CAG) or percutaneous coronary intervention (PCI) that can lead to emergency surgical aortic repair, coronary artery bypass graft surgery (CABG) or death.^1,2^ According to the extent of aortic root involvement, Dunning et al.^3^ proposed three classes of aortocoronary dissection: Class I for focal dissection limited to the sinus of Valsalva; Class II for dissection that propagated less than 40 mm to the ascending aorta; and Class III for dissection extending 40 mm or more to the ascending aorta. Management of this rare entity is still technically challenging and the optimal treatment of aortocoronary dissection complicating CAG or PCI had not been clearly established. In this article, we describe a case series of aortocoronary dissection among consecutive diagnostic CAG and PCI procedures, which were successfully treated by coronary ostial stenting.

### Case 1

**Figure 1:**
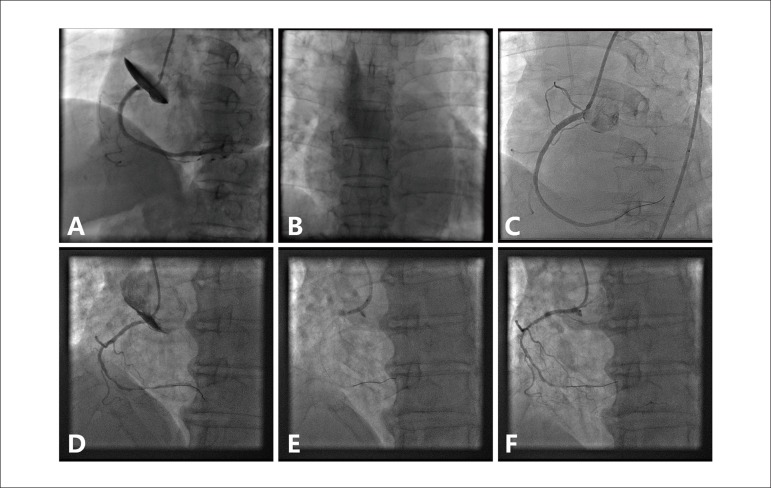
(A) Angiogram demonstrating proximal right coronary artery dissection, extending to sinus of Valsalva and ascending aorta. (B) Persistent contrast staining was observed along the aortic wall of the ascending aorta. (C) Stenting of the ostium of the right coronary artery to coverage the entry point of the dissection and final angiogram showed complete coverage of the aortocoronary dissection. (D) Angiogram showing a dissection ostium of the right coronary artery, extending retrogradely into the sinus of Valsalva and ascending aorta. (E) Stent deployment aiming at full sealing of the entry site of dissection and the RCA ostium. (F) Angiogram after ostial stenting revealed the dissection limited to the sinus of Valsalva

A 62-year-old woman with unstable angina pectoris was referred for diagnostic CAG at a local hospital. The left coronary system appeared normal. During the angiography of the right coronary artery (RCA), a coronary dissection progressing retrogradely more than 40 mm (Dunning dissection class III) from the proximal RCA into the Valsalva sinus and the ascending aorta occurred (Figure 1A and 1B). The patient suddenly complained of severe chest pain and developed hypotension and bradycardia. She was transferred immediately to our cath lab for the management of aortocoronary dissection. We decided to perform ostial stenting to seal the entry site of dissection and to stop blood flow into the false lumen. A Runthrough guidewire (Terumo, Japan) was rapidly advanced into the distal RCA. After pre-dilation with a Maverick 2.0×20 mm balloon (Boston Scientific, USA), a 3.0×24 mm stent (PROMUS Element, Boston Scientific, USA) was immediately deployed at the RCA ostium to cover the presumed entry-door of the dissection. Repeated angiography revealed no contrast leakage into the ascending aorta false lumen (Figure 1C). A computed tomography angiography (CTA) performed immediately after the PCI demonstrated complete halt of the dissection without progression. The patient had an uneventful hospital stay and repeated CTA performed two weeks later showed complete resolution of the aortic hematoma.

### Case 2

A 60-year-old man with hypertension and diabetes was admitted with effort angina for four months. CAG showed severe stenosis of the distal RCA. Transradial PCI was performed using a 5F JR4.0 guiding catheter (Cordis, USA) and Rinato guidewire (Terumo, Japan). The distal lesion was predilated using Maverick 2.0×15 mm balloon (Boston Scientific, USA). Immediately after removing the balloon, the patient complained of anterior chest pain and back pain. Angiography revealed a dissection at the RCA ostium, extending retrogradely into the sinus of Valsalva and ascending aorta (Dunning dissection class II) (Figure 1D). A 2.5×24 mm stent (EXCEL, JW Medical System, China) was immediately implanted at the RCA ostium to cover the entry point and RCA ostium, followed by post-dilatation (Figure 1E). After stenting, angiogram demonstrated no further extravasation of contrast medium (Figure 1F). An emergent CTA scan showed a limited intramural hematoma of ascending aorta. The clinical course was uneventful. At two-month follow-up, control CTA showing total resolution of the intramural hematoma.

### Case 3

**Figure 2:**
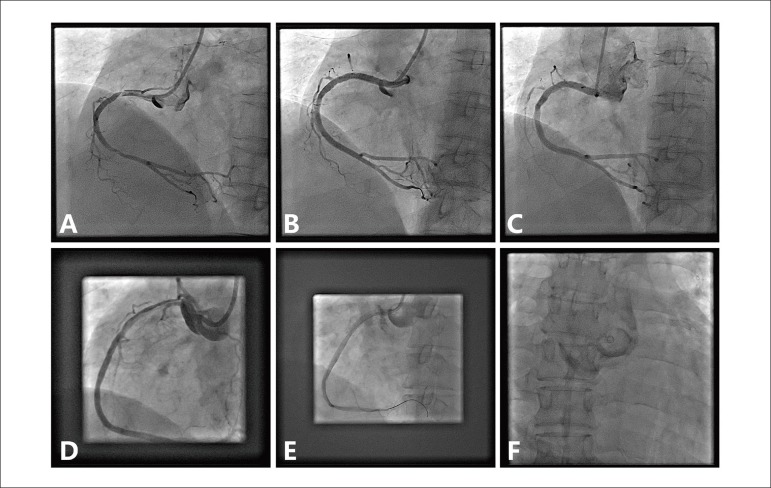
(A) After pre-dilation, a dissection of the proximal right coronary artery extending retrogradely into the sinus of Valsalva occurred. (B) Repeated angiogram after stenting demonstrating the aortic dissection was successfully sealed and limited in the sinus of Valsalva. (C) Follow-up angiogram showing complete resolution of the dissection. (D) After contrast injection, a dissection of the proximal right coronary artery with propagation into the aortic sinus and ascending aorta developed. (E) After right coronary artery ostium stenting, angiogram showed no further contrast leakage from the ostium entry point of the right coronary point to the false lumen of the ascending aorta. (F) Persistent contrast dye present in the wall of ascending aorta.

A 63-year-old woman with hypertension was admitted for effort angina of 2 week duration. CAG revealed proximal and mid-RCA diffused stenosis of approximately 90%. Transradial PCI was performed with a 6F JL 3.5 guiding catheter and a Runthrough guidewire. After pre-dilation with a 2.0 mm balloon, a 3.0 mm × 36 mm stent (Partner, Lepu Medical Technology, China) was successfully implanted in the mid-RCA. To prepare for stenting of the proximal RCA, we used the stent balloon of the mid-RCA to dilate the proximal portion at 14atm. However, after pre-dilation of the proximal lesion, a proximal RCA dissection occurred, which extended antegradely and also retrogradely into the Sinus of Valsalva (Dunning dissection class I) (Figure 2A). A 3.5×29 mm stent (Partner, Lepu Medical Technology, China) was immediately deployed at the proximal RCA, which was considered as the entry point of the aortic dissection. The final angiogram showed the aortic dissection was limited in the sinus of Valsalva (Figure 2B). A follow-up angiography was performed one week later and it revealed no residual contrast staining of the aortic wall (Figure 2C). The patient remained asymptomatic for one month without any clinical event.

### Case 4

A 52-year-old woman with hypertension and hyperlipidemia presented with chest discomfort for one week. CAG demonstrated critical stenosis in the proximal and mid-portion of the RCA. During PCI, the RCA ostium was engaged with a 6F Amplatzer left1 (Cordis, USA) guiding catheter. Before attempting to advance the guidewire, contrast medium was injected. Immediately after the injection, a large proximal RCA dissection occurred, which quickly extended in a retrograde fashion into the ascending aorta (Figure 2D). Despite obliteration of the RCA dissection with a PROMUS Element 3.0×38 mm stent, persistent dye staining of the ascending aorta was present on final angiogram (Dunning dissection class III) (Figures 2E and 2F). A computed tomography scan performed 12 hours later showed an intramural hematoma ascending aorta without an intimal flap. The patient recovered well after stenting and was discharged 7 days later. At the one-month follow-up, the patient was asymptomatic and the CTA showing complete healing of the dissection.

## Discussion

Iatrogenic aortocoronary dissection complicating coronary interventions is extremely rare and a few cases have been reported. The incidence of this complication was approximately 0.02% for diagnostic coronary angiography and 0.02-0.83% for PCI procedures.^2-5^ The rapid propagation of aortocoronary dissection may become immediately life threatening via several mechanisms, including hemorrhage into the pericardium resulting in cardiac tamponade, occlusion of the contralateral coronary ostium, or propagation of the dissection into the descending aorta.^6,7^ Most reported iatrogenic aortocoronary dissections have been related to procedures in the RCA, especially during PCI for chronic total occlusions.^2^ The RCA is more easily dissected retrogradely into the coronary sinus than the left main coronary artery (LMCA). This may be because the periostial wall and sinotubular junction of the LMCA are formed by more smooth muscle cells and by a dense matrix of collagen type I fibers^8^.

Its mechanism involves disruption of the coronary intimal by mechanical trauma, followed by vigorous contrast injection, which, in turn, contributes to subsequent retrograde extension of the dissection. The entry port is usually created by direct trauma from the catheter tip, forceful balloon inflation, the dilation of a calcified plaque, from aggressive manipulation of rigid or hydrophilic guide wires, or vigorous contrast injection with a wedged catheter.^1,9,10^ In the present cases, the cause of dissection in case 1 and case 4 was thought to be noncoaxial engagement of the catheter followed by continuous vigorous contrast injection. In case 2, the trigger for the dissection was thought to be direct trauma caused by the tip of the guiding catheter, whereas in case 3, the dissection may have been caused by dilation of the balloon in the proximal RCA, with its propagation into the ostium and the coronary sinus of Valsalva.

To date, the optimal treatment of this rare entity has not been well established. Several methods including emergent surgery, coronary artery stenting, or conservative medical treatment have been proposed to manage aortocoronary dissection.^11-13^ Given that over 40% of the cases will spread rapidly to the ascending aorta if the entry-door is not sealed rapidly, a “wait and see” approach may be too risky for uncontrollable dissection and major complications.^9^ Therefore, once aortocoronary dissection occurred, every effort should be undertaken to prevent rapid progression of the dissection. Dunning et al.^3^ proposed that patients might be successfully managed by stenting of the entry point of the coronary dissection if the dissection extends < 40 mm from the coronary ostium and that surgical intervention might be required if the dissection extends > 40 mm from the ostium. However, Park et al.^13^ reported a case of iatrogenic coronary dissection with extensive propagation into the entire ascending aorta complicating PCI for chronic total occlusion of the RCA, which was successfully managed by stenting of the RCA ostium with favorable outcome. Carstensen and Ward^9^ reviewed 67 cases published in the literature and suggested that even rapidly propagating dissections can be successfully managed percutaneously and that attempting to halt propagation does not appear to compromise the chances of surgical success if the initial approach fails. Additionally, the surgical repair of catheter-related dissection may be more risky in the setting of coronary ischemia and following PCI with full anticoagulation and antiplatelet therapy. Boukhris et al.^2^ recently assessed the management strategy and outcomes of such a complication among 956 cases of complicating PCI for chronic total occlusion, and found aortocoronary dissection occurred in eight patients for an overall frequency of 0.83%. In all these cases, rapid ostial stenting was performed and no emergency surgery was required. In Shorrock et al^1^ study, four of six patients (67%) with aortocoronary dissection were treated with ostial stenting, one underwent emergency CABG, and the remaining one was treated conservatively without subsequent adverse clinical outcomes. Moreover, they performed a systematic literature review of 107 published cases of aortocoronary dissection during PCI, and showed that this complication were most commonly treated with stenting (49.5%) or conservative management (21.5%) although approximately 29% required surgery. Hence, Shorrock et al.^1^ proposed that emergency surgery for aortocoronary dissection is not needed in the vast majority of cases and should only be considered in cases of occlusion of the dissected vessel with cessation of antegrade flow that cannot be restored percutaneously, and extension of the dissection to the descending aorta.

In our case series, rapid ostial stenting was performed in all of them, and all patients had uneventful recovery. Follow-up imaging with CTA or CAG showed complete resolution of the dissection in all patients. So, the outcome of coronary stenting for the management of aortocoronary dissection is relatively favorable.

It is worth noting that the best approach to management of aortocoronary dissection is to prevent its occurrence. During catheterization, there should be a coaxial alignment of the catheter with the coronary artery, and meticulous attention should be paid to the pressure waveform. Once the pressure waveform is dampened, the contrast should not be injected. Moreover, if aortocoronary dissection occurs, stopping antegrade contrast injections is critical to avoid propagation and enlargement of the dissection^12^. In addition, a careful and gentle handling of wires and guiding catheter probably would prevent some of the cases of this catastrophic complication.

## Conclusion

Iatrogenic aortocoronary dissection is an infrequent but life-threatening complication of diagnostic CAG and PCI, necessitating a prompt diagnosis and appropriate treatment. Immediate coronary ostial stenting to seal off the entry point of the dissection is a feasible and reasonable initial management for this devastating complication.
